# Dapagliflozin Inhibits Cell Adhesion to Collagen I and IV and Increases Ectodomain Proteolytic Cleavage of DDR1 by Increasing ADAM10 Activity

**DOI:** 10.3390/molecules25030495

**Published:** 2020-01-23

**Authors:** Junichi Okada, Eijiro Yamada, Tsugumichi Saito, Hideaki Yokoo, Aya Osaki, Yoko Shimoda, Atsushi Ozawa, Yasuyo Nakajima, Jeffrey E. Pessin, Shuichi Okada, Masanobu Yamada

**Affiliations:** 1 Division of Endocrinology and Metabolism, Graduate School of Medicine, Gunma University, 3-39-15 Showa-machi, Maebashi, Gunma 371-8511, Japan; m1720012@gunma-u.ac.jp (J.O.); eijiro.yamada@gunma-u.ac.jp (E.Y.); saitotsu@gunma-u.ac.jp (T.S.); ashibata@gunma-u.ac.jp (A.O.); m13702015@gunma-u.ac.jp (Y.S.); ozawaa@gunma-u.ac.jp (A.O.); ynihei@gunma-u.ac.jp (Y.N.); myamada@gunma-u.ac.jp (M.Y.); 2 Department of Human Pathology, Graduate School of Medicine, Gunma University, 3-39-15 Showa-machi, Maebashi, Gunma 371-8511, Japan; hyokoo@gunma-u.ac.jp; 3 Department of Medicine and Molecular Pharmacology, Albert Einstein College of Medicine, Bronx, New York, NY 10461, USA; jeffrey.pessin@einstein.yu.edu

**Keywords:** sodium-glucose cotransporter 2 inhibitor, ADAM10, diabetes mellitus, discoidin domain receptor, colon cancer

## Abstract

Dapagliflozin, empagliflozin, tofogliflozin, selective inhibitors of sodium-glucose cotransporter 2 (SGLT2), is used clinically to reduce circulation glucose levels in patients with type 2 diabetes mellitus by blocking the reabsorption of glucose by the kidneys. Dapagliflozin is metabolized and inactivated by UGT1A9. Empagliflozin is metabolized and inactivated by UGT1A9 and by other related isoforms UGT2B7, UGT1A3, and UGT1A8. Tofogliflozin is metabolized and inactivated by five different enzymes CYP2C18, CYP3A4, CYP3A5, CYP4A11, and CYP4F3. Dapagliflozin treatment of HCT116 cells, which express SGLT2 but not UGT1A9, results in the loss of cell adhesion, whereas HepG2 cells, which express both SGLT2 and UGT1A9, are resistant to the adhesion-related effects of dapagliflozin. PANC-1 and H1792 cells, which do not express either SGLT2 or UGT1A9, are also resistant to adhesion related effects of dapagliflozin. On the other hand, either empagliflozin or tofogliflozin treatment of HCT116, HepG2, PANC-1, and H1792 cells are resistant to the adhesion-related effects as observed in dapagliflozin treated HCT116 cells. Knockdown of UGT1A9 by shRNA in HepG2 cells increased dapagliflozin sensitivity, whereas the overexpression of UGT1A9 in HCT116 cells protected against dapagliflozin-dependent loos of cell adhesion. Dapagliflozin treatment had no effect on cellular interactions with fibronectin, vitronectin, or laminin, but it induced a loss of interaction with collagen I and IV. In parallel, dapagliflozin treatment reduced protein levels of the full-length discoidin domain receptor I (DDR1), concomitant with appearance of DDR1 cleavage products and ectodomain shedding of DDR1. In line with these observations, unmetabolized dapagliflozin increased ADAM10 activity. Dapagliflozin treatment also significantly reduced Y792 tyrosine phosphorylation of DDR1 leading to decrement of DDR1 function and detachment of cancer cells. Concomitant with these lines of results, we experienced that CEA in patients with colon cancer, which express SGLT2 but not UGT1A9, and type 2 diabetes mellitus treated by dapagliflozin in addition to chemotherapy was decreased (case 1). CEA in patients with colon cancer, which express SGLT2 but not UGT1A9, and type 2 diabetes mellitus was treated by dapagliflozin alone after radiation therapy was decreased but started to rise after cessation of dapagliflozin (case 2). CA19-9 in two of patients with pancreatic cancer and type 2 diabetes mellitus was resistant to the combination therapy of dapagliflozin and chemotherapy (case 3 and 4 respectively). PIVKAII in patients with liver cancer and type 2 diabetes mellitus, and CYFRA in patients with squamous lung cancer and type 2 diabetes mellitus was also resistant the combination therapy of dapagliflozin and chemotherapy (case 5 and 6 respectively). Taken together, these data suggest a potential role for dapagliflozin anticancer therapy against colon cancer cells that express SGLT2, but not UGT1A9.

## 1. Introduction

In mammals, there are two major families of glucose transporters. The first family contains glucose transporter (GLUT) proteins, which are stereospecific facilitative glucose transporters, with net transport driven by the concentration gradient of glucose across the membrane [1]. The second family contains sodium-dependent glucose cotransporters (SGLTs) [2], which use the sodium concentration gradient to drive the uptake of glucose, allowing for more efficient uptake and net flux against the glucose concentration gradient [2]. Over the past several years, selective inhibitors of SGLT2 (SGLT2i) have been used as effective therapeutic agents to prevent the reabsorption of kidney-filtered glucose, and they are widely used for treatment of hyperglycemia in patients with diabetes mellitus [2]. Recently, SGLT2i has been reported to possess nephroprotection action against nephropathy [3,4] and a potential to treat heart failure [5,6].

It is well established that tumor cells typically undergo a metabolic switch towards utilization of glucose and away from fatty acid oxidation [7]. However, cancer cells express many types of GLUTs to maintain the high levels of glucose needed for their high metabolic rates and to provide critical substrates for DNA and RNA synthesis that are not available through fatty acid oxidation [2]. As such, GLUT inhibitors have been considered as anticancer drug therapies [7]. However, inhibiting global glucose transport would be challenging, as healthy cells require the function of GLUT family transporters to maintain normal biological activity [1,2]. In addition to these issues, as cancer cells can reprogram the metabolic condition between glucose metabolism, lipid metabolism, and amino acid metabolism, simply suppressing glucose uptake into cancer cells is unlikely to be a promising approach to treat cancer [8].

SGLT2 expression is generally restricted to the proximal tubules of the kidneys [2] and SGLT2i has already been proven safe in humans [2]. SGLT2 is also expressed in various cancer cells [2] and we recently reported that treatment with SGLT2i dapagliflozin reduced cultured cancer cell number without affecting apoptosis or cell growth [9]. A distinctive feature of dapagliflozin compared to other SGLT2is is its conjugation to glucuronic acid by UDP Glucuronosyltransferase Family 1 Member A9 (UGT1A9), which both inactivates dapagliflozin and induces its excretion [1,2,7,10]. Similarly, empagliflozin is inactivated and excreted by UGT1A9 but also by UDP Glucuronosyltransferase Family 2 Member B7 (UGT2B7), UDP Glucuronosyltransferase Family 1 Member A3 (UGT1A3) and UDP Glucuronosyltransferase Family 1 Member A8 (UGT1A8). In addition, tofogliflozin is inactivated and excreted by five different enzymes (cytochrome P450 family 2 subfamily C member 18 (CYP2C18), cytochrome P450 family 3 subfamily A member 4 (CYP3A4), cytochrome P450 family 3 subfamily A member 5 (CYP3A5), cytochrome P450 family 4 subfamily A member 11 (CYP4A11), and cytochrome P450 family 4 subfamily F member 3 (CYP4F3)). In this study, we have uncovered a novel physiological action of dapagliflozin in the suppression of cancer cell adhesion through activation of ectodomain shedding of discoid domain receptor 1.

## 2. Materials and Methods

### 2.1. Reagents

Dapagliflozin (CS-0781), empagliflozin (AG-CR1-3619-M-010), and tofogliflozin (CS-3793), antibody recognizing DDR1 (GTX111453), were purchased from Funakoshi Co., Ltd (Tokyo, Japan). Anti-SGLT2 antibody (sc-393350) was purchased from Santa Cruz Biotechnology (Dallas, TX, USA). Phospho-Y792 DDR1 antibody (11994) was purchased from Cell Signaling Technology (Danvers, MA, USA). UGT1A9 (ab96214) was purchased from Abcam (Cambridge, UK). Horseradish peroxidase (HRP)-conjugated anti-rabbit (G-21234) and HRP-conjugated anti-mouse (G-21040) IgG antibodies were obtained from Thermo Fisher Scientific (Waltham, MA, USA). Cell culture medium and reagents were purchased from Thermo Fisher Scientific. The CytoSelect 48-well cell adhesion assay kit (CBA-070) was purchased from Cell Biolabs (San Diego, CA, USA). shRNA for UGT1A9 (SR310169) was purchased from OriGene (Rockville, MD, USA). The SensoLyte^®^ 520 ADAM10 Activity Assay Kit (AS-7226) was obtained from AnaSpec (Fremont, CA, USA). All other chemicals used in this study were purchased from Sigma-Aldrich (St. Louis, MO, USA).

### 2.2. Cell Culture

The HCT116 (Human colon cancer cell line; CCL-247), HepG2 (Human hepatic cancer cell line; HB-8065), PANC-1 (Pancreatic cancer cell line; CRL-1469), and H1792 (Lung cancer cell line; CRL-5895) cells were purchased from American Type Culture Collection (ATCC) (Manassas, VA, USA). The HCT116 cells were maintained in McCoy’s 5A medium with 10% fetal bovine serum, and HepG2, PANC-1 cells were maintained in Dulbecco’s modified Eagle’s medium supplemented with 10% fetal bovine serum. The H1792 cells were maintained in RPMI medium with 10% fetal bovine serum. The cells were grown to subconfluence and incubated with either DMSO or dapagliflozin, at concentrations indicated in the figure legends. The cells were frozen using liquid nitrogen and kept at −80 °C until further use.

### 2.3. Transfections

HCT116 and HepG2 cells were electroporated with a total of 300 μg of plasmid DNA at 950 microfarads and 0.2 kV, as described previously [11]. Under these conditions, 70% of the cells were functionally transfected, as determined by in situ staining for β-galactosidase activity.

### 2.4. Immunoblotting

Scraped frozen cells were rocked for 10 min at 4 °C with NP-40 lysis buffer (25-mM Hepes, pH 7.4, 10% glycerol, 50-mM sodium fluoride, 10-mM sodium pyrophosphate, 137-mM sodium chloride, 1-mM sodium orthovanadate, 1-mM PMSF, 10-μg/mL aprotinin, 1-μg/mL pepstatin, and 5-μg/mL leupeptin). Insoluble material was separated from the soluble extract by centrifugation, the total protein in the supernatant was quantified by the BCA method, and the samples were normalized by total protein content. The samples were resuspended in SDS sample buffer and heated at 100 °C for 5 min. Samples were separated by SDS polyacrylamide gel electrophoresis (SDS-PAGE, 18 × 16 cm) and electrophoretically transferred to polyvinylidene difluoride (PVDF) membranes. The samples were immunoblotted with specific antibodies, as indicated in the figure legends.

### 2.5. Cell Adhesion Assays

Changes in cell adhesion induced by dapagliflozin were estimated with the Cell Biolabs CytoSelectTM Cell Adhesion assay kit following the manufacturer’s instructions. Cultured cells were incubated with or without 2-mM dapagliflozin for 20 min for the assay.

### 2.6. Shedding Activity Assays

Purified ADAM10 and substrate were obtained by SensoLyte 520 ADAM10 activity assay kit. Supplied ADAM10 (10 μg), supplied substrate (5 μM), and dapagliflozin (80 μg) were mixed with supplied buffer and incubated for 60 min at room temperature. The resulting florescence was measured at 520 nM.

### 2.7. Statistical Analyses

All data in figures are expressed as means ± standard deviation. Data were analyzed using one-factor ANOVA to compare the means of all groups. The Tukey–Kramer multiple comparisons procedure from the InStat 2.00 program was used to determine statistical differences between the means. A *p* value of <0.05 was considered statistically significant.

### 2.8. Compliance with Ethics Guidelines

The study protocol was reviewed and approved by the review board of Gunma University in accordance with the principles of the Declaration of Helsinki.

## 3. Results

### 3.1. Relative Sensitivities of Several Tumor Cell Lines (HCT116, HepG2, PANC-1, and H1792) to the SGLT2 Inhibitors, Dapagliflozin, Empagliflozin, and Tofogliflozin

**Figure 1:**
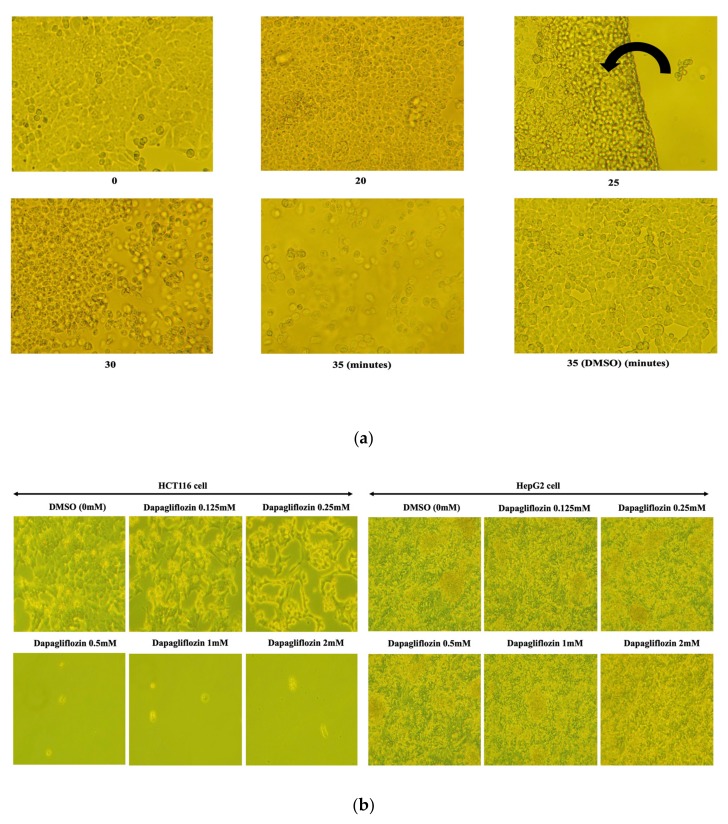

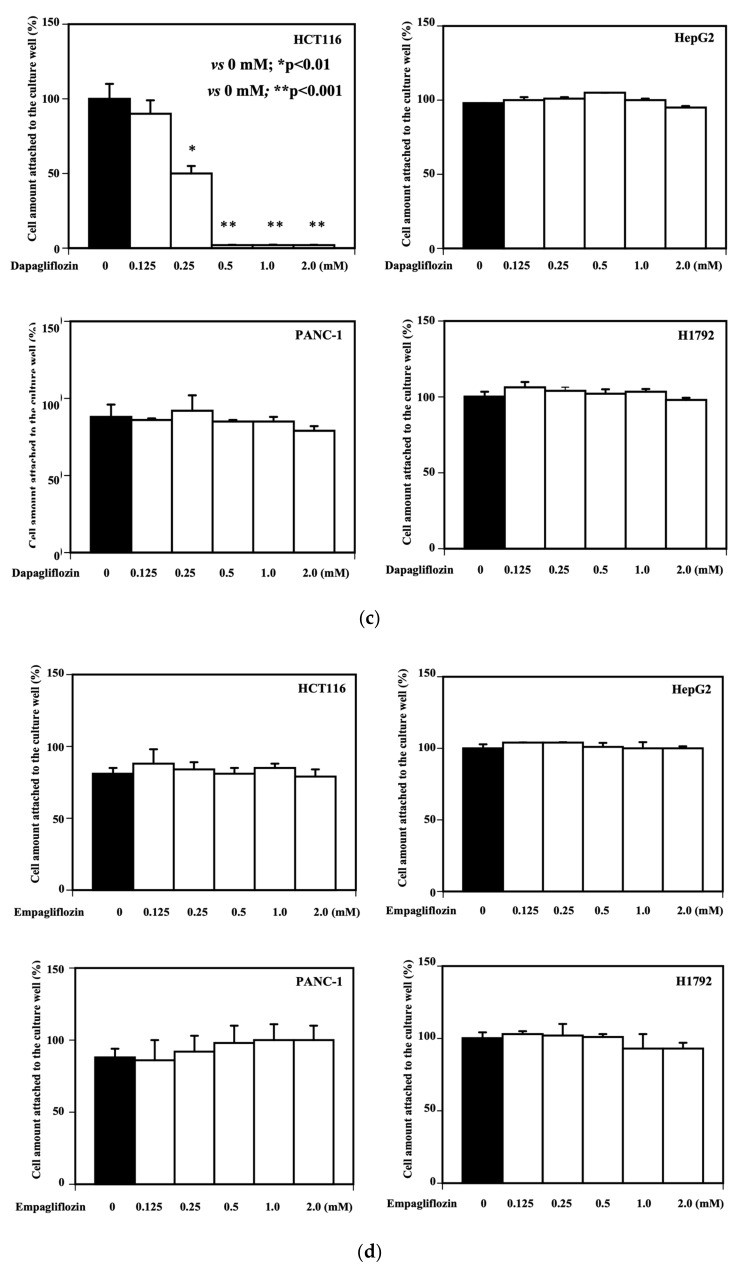

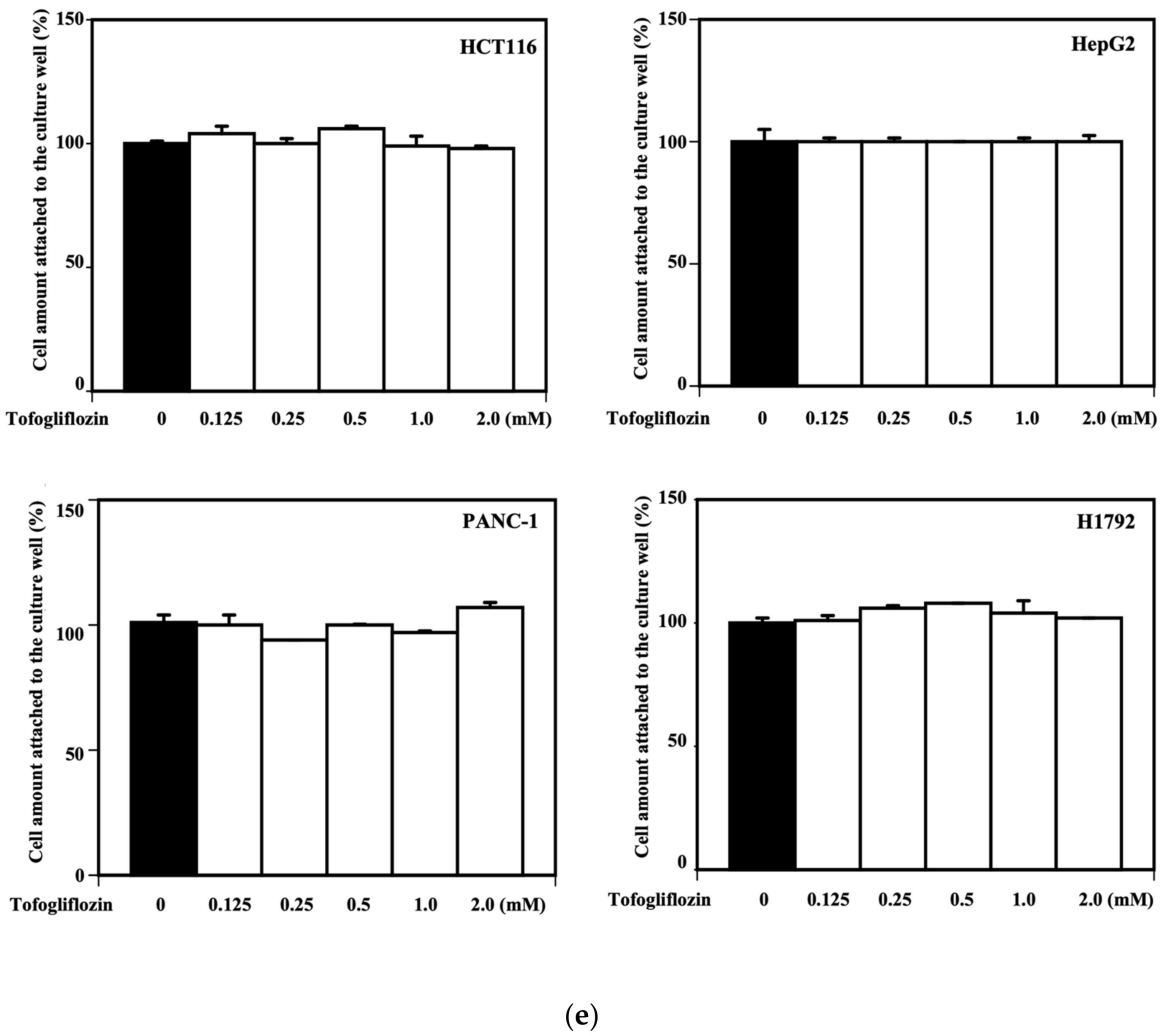
Relative sensitivities of HCT116 and HepG2 cells to dapagliflozin treatment. (**a**) Time-course effects of dapagliflozin treatment on HCT116 cell morphology and cell attachment. The HCT116 cells were treated with vehicle (DMSO) or 0.5 mM dapagliflozin for the times indicated. Please note that 25 min treatment with 0.5 mM dapagliflozin let HCT116 cells be lifted off the dish as a sheet and flipped over onto the side of the plate, as indicated by the arrow. This phenomenon suggested us that the cell attachment was impaired by dapagliflozin treatment. Phase-contrast microscopy images (×100 magnification) are presented. These experiments were conducted in triplicate, and the typical results are shown. (**b**) (**left panel**) HCT116 cells were treated with either vehicle (DMSO) or 0.125, 0.25, 0.5, 1.0, or 2.0 mM dapagliflozin for 35 min. The experiments were conducted independently in triplicate, and typical results are presented (×100 magnification). (**b**) (**right panel**) HepG2 cells were treated with either vehicle (DMSO) or 0.125, 0.25, 0.5, 1.0, or 2.0 mM dapagliflozin for 35 min. The experiments were conducted independently in triplicate, and typical results are presented (×100 magnification). (**c**) Effect of dapagliflozin on HCT116, HepG2, PANC-1, and H1792 cells were quantified and presented as a bar graph. The *Y*-axis represents the amounts of cells attached to the culture well and the *X*-axis represents the concentration of dapagliflozin added to the culture medium (n = 3, in each column, 0 mM vs. 0.125 mM; not significant, 0 mM vs. 0.25 mM; * *p* < 0.01, 0 mM vs. 0.5, 1.0, 2.0 mM; ** *p* < 0.001). (**d**) Effect of empagliflozin on HCT116, HepG2, PANC-1, and H1792 cells were quantified and presented as a bar graph. The *Y*-axis represents the amounts of cells attached to the culture well and the *X*-axis represents the concentration of empagliflozin added to the culture medium (n = 3). (**e**) Effect of tofogliflozin on HCT116, HepG2, PANC-1, and H1792 cells were quantified and presented as a bar graph. The *Y*-axis represents the amounts of cells attached to the culture well and the *X*-axis represents the concentration of tofogliflozin added to the culture medium (n = 3).

Based upon our previous findings [9], we first treated HCT116 cells with 0.5 mM dapagliflozin for various time periods (Figure 1a).

As shown in Figure 1a, the first indication of cell detachment was observed after 25 min, and most cells had detached by 35 min. This did not occur when the HCT116 cells incubated with the vehicle (DMSO) used to dissolve dapagliflozin (Figure 1a). We next examined the dapagliflozin dose response effect on both HCT116 and HepG2 cells (Figure 1b).

After 35 min of incubation, HCT116 cells treated with 0.125 mM dapagliflozin began to display morphological changes associated with cell detachment (Figure 1b, left panel), whereas cells treated with 0.5 mM dapagliflozin were almost completely detached from the substratum (Figure 1b, left panel). These results were quantified and are represented in Figure 1c (HCT116 upper panel in left side). In contrast, HepG2 cells were unaffected by the dapagliflozin treatment up to 2 mM (Figure 1b, right panels and upper panel in right side in Figure 1c). We also tested dapagliflozin’s effects on a pancreatic cancer cell line (PANC-1) and lung cancer cell line (H1792). As shown in Figure 1c, dapagliflozin did not appear to have any effect on the two cell lines (PANC-1; lower panel in left side and H1792; lower panel in right side). Similarly, two other SLGT2 inhibitors, empagliflozin and tofogliflozin had no significant effect on HCT116, HepG2, PANC-1 or H1792 cells (Figure 1d,e). Taken together, these data demonstrate that dapagliflozin treatment results in a loss of cell adherence and that HCT116 cells are more sensitive to dapagliflozin treatment than HepG2, PANC-1, and H1792 cells. These data also demonstrate that the loss of cell adhesion is selective to dapagliflozin as two other SGLT2 inhibitors (empagliflozin and tofogliflozin) do not possess this activity.

### 3.2. Sensitivity of HCT116 and HepG2 Cells to Dapagliflozin is Dependent on SGLT2 and UGT1A9 Protein Levels

**Figure 2:**
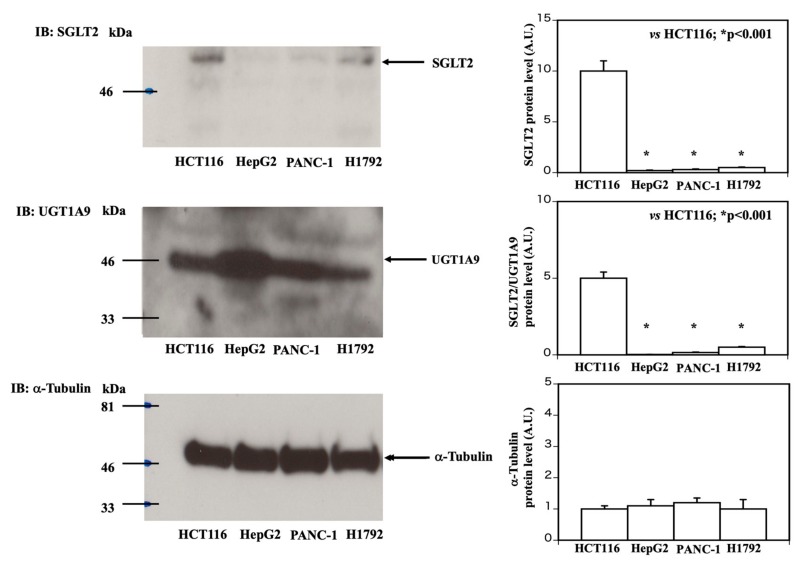
Comparison of SGLT2 and UGT1A9 protein levels in HCT116, HepG2, PANC-1, and H1792 cells. HCT116, HepG2, PANC-1, and H1792 cell extracts were prepared and immunoblotted for SGLT2 (upper panel on left side), UGT1A9 (middle panel on left side), and alpha-tubulin (as loading control; lower panel on left side). The quantification of the SGLT2 protein levels (upper panel on the right side, n = 3, HCT116 vs. HepG2 or PANC-1 or H1792, * *p* < 0.001), SGLT2 protein levels against UGT1A9 protein levels (middle panel on the right side, n = 3, HCT116 vs. HepG2 or PANC-1 or H1792, * *p* < 0.001), and alpha-tubulin protein levels (lower panel on the right side, not significant among four cells) is shown as a bar graph.

To investigate the mechanisms by which dapagliflozin treatment causes cell detachment only in the HCT116 cells, we examined differences among the HCT116, HepG2, PANC-1, and H1972 cells that could be related to their different responses to dapagliflozin. Immunoblotting indicated that the HCT116 cells (Figure 2, upper panel) expressed approximately five times more SGLT2 protein than the H1792 cells and approximately ten times more SGLT2 protein than HepG2 and PANC-1 cells. These results were quantified and are represented by the bar graph on the right side. In contrast, the HepG2 cells expressed approximately 20 times more UGT1A9 protein than the HCT116, PANC-1, and H1792 cells (Figure 2, middle panel). These results were quantified and are represented by the bar graph on the right side. Alpha-tubulin was used as a loading control for the comparisons (Figure 2, lower panel). These results were quantified and are represented by the bar graph on the right side.

**Figure 3:**
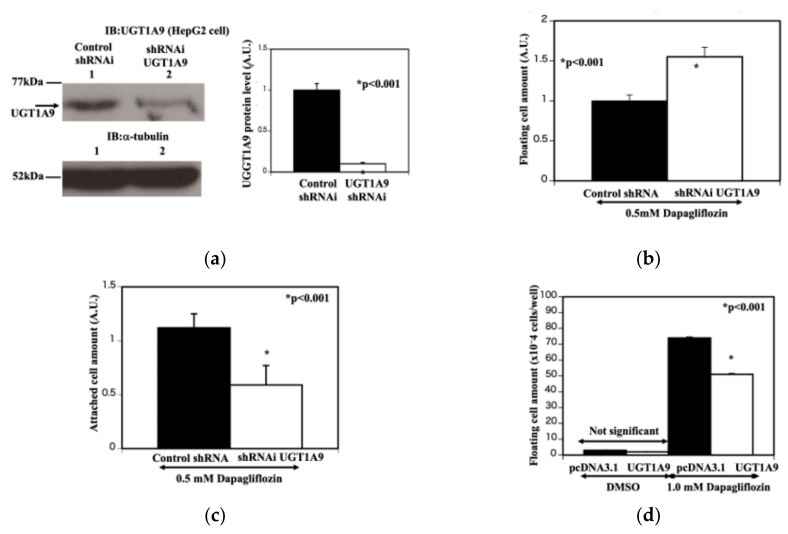
UGT1A9 protein level is critical for susceptibility of dapagliflozin in HepG2 and HCT116 cells. (**a**) Knockdown of UGT1A9. HepG2 cells were transfected with either control shRNAi (lane 1) or UTG1A9-specific shRNA (lane 2). Cell extracts were prepared and immunoblotted for UTG1A9 (upper panel) and alpha-tubulin (lower panel). The quantification of the relative amounts of UTG1A9 protein levels compared with alpha-tubulin is shown as a bar graph (n = 3, control shRNAi vs. UGT1A9-specific shRNA, * *p* < 0.001). The *Y*-axis represents the UGT1A9 protein level. The closed column represents control shRNAi-treated HepG2 cells and the open column represents UTG1A9 specific shRNA-treated HepG2 cells. (**b**) Estimation of floating cell amount (HepG2 cells). The control shRNAi-treated and UTG1A9-specific shRNA-treated HepG2 cells were treated with 0.5 mM dapagliflozin for 35 min. The culture supernatants were collected and centrifuged at 2000 g for 5 min. The cell pellets were resuspended in 1 mL PBS, and the protein contents of the pellets were determined to indicate the amounts of floating cells in the culture following dapagliflozin treatment (n = 3, control shRNAi vs. UGT1A9-specific shRNA, * *p* < 0.001). The closed column represents control shRNAi-treated HepG2 cells and the open column represents UTG1A9 specific shRNA-treated HepG2 cells. (**c**) Estimation of remained cell amount on the well (HepG2 cells). The control shRNAi-treated and UTG1A9-specific shRNA-treated HepG2 cells were treated with 0.5 mM dapaglifloin for 35 min. After the culture supernatant was removed, the cells remaining on the culture plate were trypsinized and resuspended in PBS. The cell amount remaining on the culture plate after dapagliflozin treatment was determined via a protein assay (n = 3, control shRNAi vs. UGT1A9-specific shRNA, * *p* < 0.001). The closed column represents control shRNAi-treated HepG2 cells and the open column represents UTG1A9-specific shRNA-treated HepG2 cells. (**d**) Estimation of floating cell amount with UGT1A9 overexpression (HCT116 cells). HCT116 cells were transfected with empty pcDNA3.1 plasmid or pcDNA3.1 containing the UGT1A9 coding sequence. The cells were then treated with vehicle or 1.0 mM dapagliflozin for 35 min. The culture medium was collected and centrifuged for 5 min at 2000 g, and the cell pellet was resuspended in 1 mL PBS. The cell amount in the pellet was estimated by a protein assay and is presented as a bar graph (n = 3 each, control pcDNA3.1 plasmid with 1.0 mM dapagliflozin vs. pcDNA3.1-UGT1A9 coding sequence with 1.0 mM dapagliflozin, * *p* < 0.01). The closed column represents empty plasmid pcDNA3.1-transfected cells and the open column represents cells treated with pcDNA3.1 containing the UGT1A9 coding sequence.

Since UGT1A9 degrades dapagliflozin, we next examined whether the different sensitivities of these cells to the dapagliflozin treatment were due to UGT1A9 protein levels. The UGT1A9-specific shRNA-transfected HepG2 cells (Figure 3a, lane 2) exhibited a substantial reduction of UGT1A9 protein compared with control shRNA-transfected cells (Figure 3a, lane 1). Alpha-tubulin was used as a loading control for all comparisons (Figure 3a, lane 1, 2). The reduction of UGT1A9 levels was quantified and is represented by the bar graph on the right side. The reduction of UGT1A9 protein in the HepG2 cells resulted in increased dapagliflozin sensitivity (with 0.5 mM dapagliflozin, 35-min incubation), as measured by the number of floating cells (Figure 3b) and cell detachment (Figure 3c). Conversely, an increase in UGT1A9 expression in the HCT116 cells resulted in a decrease in cell detachment following 1.0-mM dapagliflozin treatment for 35 min (Figure 3d). These results are consistent with dapagliflozin-mediated cell detachment is dependent on both SGLT2 and UGT1A9 protein levels and that cells with more SGLT2 protein but less UGT1A9 protein are more sensitive to the effects of dapagliflozin.

### 3.3. Dapagliflozin Inhibits Cell Adhesion to Collagens I and IV and Increases Ectodomain Shedding of DDR1 by Increasing ADAM10 Activity

**Figure 4:**
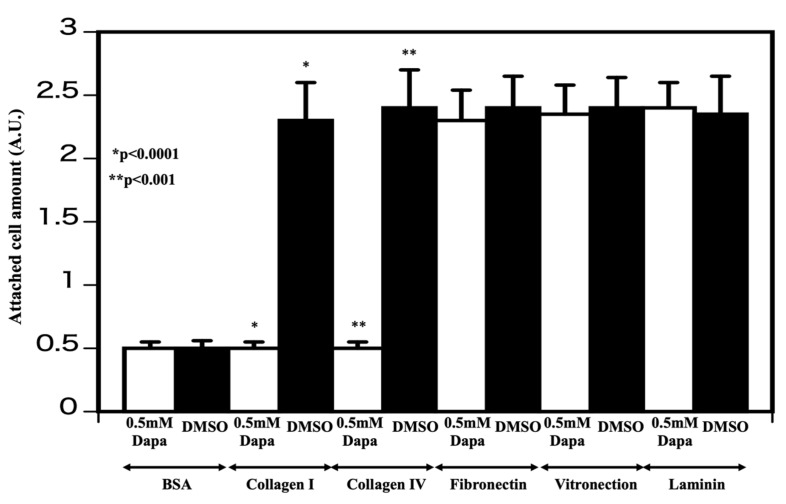
Determination of adhesion molecule affected by dapagliflozin HCT116 cells were incubated with either vehicle (DMSO) or 0.5 mM dapagliflozin for 35 min and added to tissue culture dishes coated with bovine serum albumen (BSA), collagens I and IV, fibronectin, vitronectin, or laminin. After washing, the number of cells remaining and attached to the substratum was determined as described in the Experimental procedures section (n = 3, DMSO-treated cells vs. cells treated with 0.5 mM dapagliflozin * *p* < 0.0001, ** *p* < 0.001). The open column represents cells treated with 0.5 mM dapagliflozin and the closed column represents DMSO-treated cells. The experiments were conducted independently in triplicate.

Cellular attachment to the substratum relies typically on interactions with the extracellular matrix [12]. To determine whether dapagliflozin impairs extracellular matrix protein interactions, HCT116 cells were pretreated with either DMSO or dapagliflozin (0.5 mM, 35 min) before being plated on dishes precoated with collagen I, collagen IV, fibronectin, vitronectin, or laminin. After incubating for 30 min, the cells were washed twice with PBS and stained and labeling determined with a microplate reader (absorbance was 560 nm). As shown in Figure 4, dapagliflozin selectively interfered with cell attachment to collagens I and IV, but had no significant effect on HCT116 cell attachment to fibronectin, vitronectin, or laminin. Collagens I and IV bind to DDR1 and activate their intrinsic tyrosine kinase activity, which is necessary to stimulate cell–collagen interactions [13].

**Figure 5:**
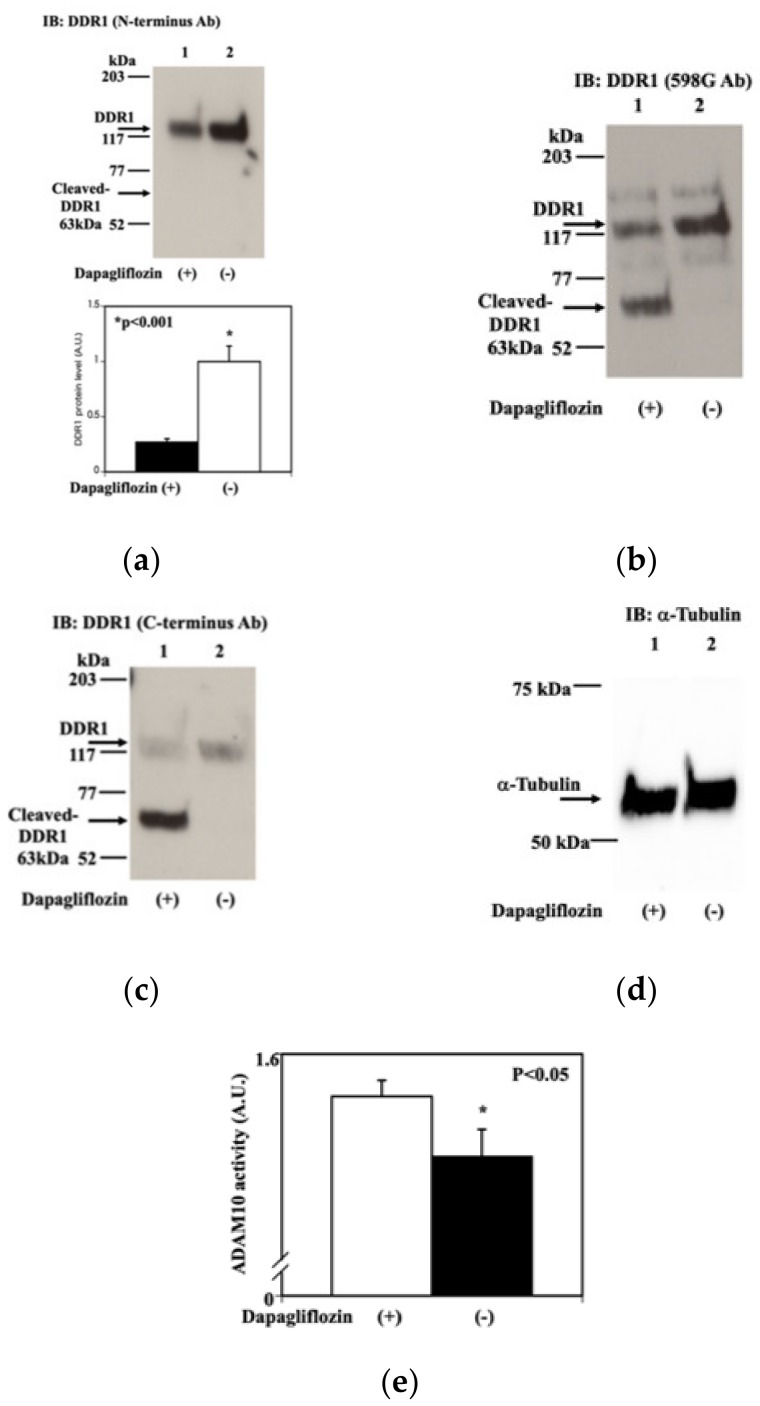
Dapagliflozin increases discoidin domain receptor 1 proteolytic cleavage. (**a**) Effect of dapagliflozin on DDR1 (N-terminus antibody). HCT116 cells were treated with either vehicle (−; lane 2) or 0.5 mM dapagliflozin (+; lane 1) for 20 min. Cell extracts were prepared and immunoblotted with an amino-terminal anti-DDR1 antibody. Only full length-DDR1 was detected. The quantification of the DRR1 protein is presented as a bar graph (n = 3, * *p* < 0.001). The closed column represents cells treated with 0.5 mM dapagliflozin and the open column represents DMSO-treated cells. The experiments were conducted independently in triplicate. (**b**) Effect of dapagliflozin on DDR1 (589G antibody). The immunoblotted membrane used in 5a was stripped and subsequently immunoblotted with 598 G antibody, which recognizes a proximal transmembrane domain epitope. Full length-DDR1 and cleaved-DDR1 showed up. (**c**) Effect of dapagliflozin on DDR1 (C-terminus antibody). The immunoblotted membrane used in Figure 5a,b was stripped and immunoblotted with a carboxy-terminal anti-DDR1 antibody. Full length-DDR1 and cleaved-DDR1 showed up as Figure 5b. (**d**) Estimation of loading amount. The immunoblotted membrane used in 5a–c was stripped and immunoblotted with anti-alpha-tubulin antibody. (**e**) Estimation of sheddase activity with or without dapagliflozin. Proteolytic activity was determined as described in the Experimental procedures section (n = 3, * *p* < 0.05). In brief, specific substrate, and dapagliflozin were mixed with the buffer and incubated for 60 min at room temperature. The resulting fluorescence was measured at 520 nM. Open column represents that the proteolytic activity was measured with 0.5 mM dapagliflozin and closed column represents that the proteolytic activity was measured with DMCO.

Therefore, we also examined whether dapagliflozin had any effect on DDR1. The immunoblotting of extracts from HCT116 cells treated with either DMSO (Figure 5a, lane 2) or dapagliflozin (0.5 mM, 20 min) (Figure 5a, lane 1) using an amino-terminal antibody demonstrated a dapagliflozin-induced decrease in the amount of full-length DDR1 protein. In contrast, immunoblotting with 598 G antibody, which recognizes the transmembrane domain (Figure 5b, lane 1), or an antibody recognizing the carboxy-terminus of DDR1 (Figure 5c, lane 1), revealed the induction of a 63-kDa fragment following dapagliflozin treatment in addition to the reduction of the amount of full-length DDR1 protein. This 63-kDa fragment was considered to be cleaved DDR1 [11]. Alpha-tubulin was used as a loading control via strip and re-probe methods for all comparisons (Figure 5d). Shedding refers to the cleavage of cell surface proteins and the subsequent release of these peptide fragments [12,13]. As DDR1 and ADAM10 consist of a complex in cancer cells [14], we determined whether unmetabolized dapagliflozin directly increases ADAM10 activity for DDR1. To do this, we established an in vitro ADAM10 activity assay, as described in the experimental procedures section. This assay demonstrated that unmetabolized dapagliflozin increases ADAM10 activity (Figure 5e). Taken together, these data indicate that unmetabolized dapagliflozin induces the cleavage of DDR1 via the activation of ADAM10 activity.

### 3.4. Dapagliflozin Reduces Y792 Tyrosine Phosphorylation of DDR1

**Figure 6:**
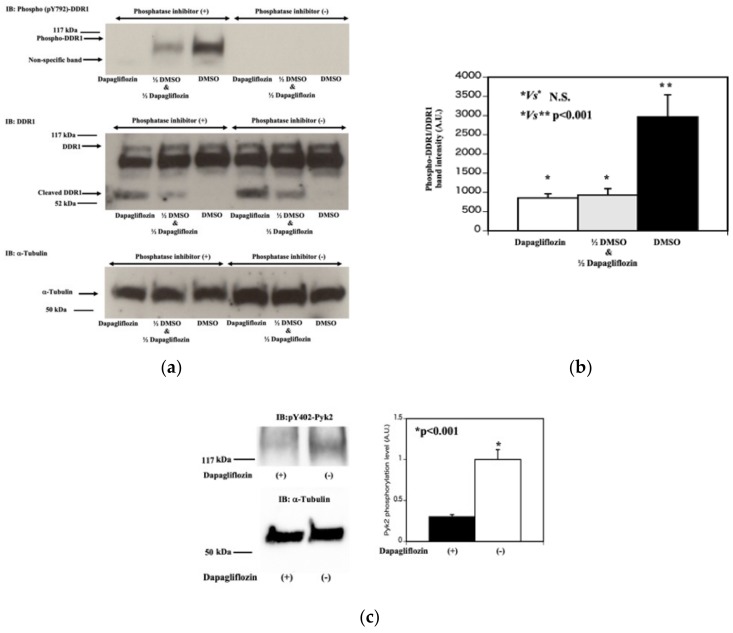
Dapagliflozin increases tyrosine phosphatase activity that reduces DDR1 dephosphorylation. (**a**) DDR1 tyrosine phosphorylation. HCT116 cells were treated with vehicle (DMSO) or 0.5 mM dapagliflozin for 20 min. The cells were extracted in buffer with (+, 1–3 lanes from the most left lane) and without (−, 4–6 lanes from the most left lane) the phosphatase inhibitors (Sodium vanadate, Sodium pyrophosphate, Sodium Fluoride). Fifty microliters of lysate from DMSO treated HCT116 cells was mixed with 50 μL lysate from dapagliflozin treated HCT116 cells and incubated for 30 min at room temperature (labeled as 1/2 DMSO and 1/2 Dapagliflozin, the second lane from the most left lane in the left upper panel). In parallel, 100 μL lysate of DMSO treated HCT116 cells (the third lane from the left lane in the upper left panel) and 100 μL lysate of dapagliflozin treated HCT116 cells (the third lane from the left lane in the left upper panel) were incubated for 30 min at room temperature. Each lysate was run on SDS-PAGE and transferred to PVDF membrane, and subjected to western blot using a pY792-DDR1 antibody (top panel), the 598 G antibody (middle panel) and α-tubulin (bottom panel). (**b**) Estimation of DDR1 tyrosine phosphorylation. Quantification of the DDR1 phosphorylation was determined by dividing the band intensity of the pY792-DDR1 antibody signal by the 598 G antibody full length DDR1 signal. (n = 3, * *p* Not significant, ** *p* < 0.001). (**c**) Effect of dapagliflozin on downstream of DDR1 signal pathway. HCT116 cells were treated with vehicle (−) or 2.0 mM dapagliflozin (+) for 20 min. Cell extracts were then immunoblotted for pY402-Pyk2 and α-tubulin. Quantification of pY402-Pyk2 relativeα-tubulin is presented in the bar graph (on the right side). (n = 3, *p* < 0.001).

After DDR1 binds to collagen I, DDR1 is auto-phosphorylated and activated [12,13,15,16]. We speculated that dapagliflozin also reduces the DDR1 tyrosine-phosphorylation. As shown in Figure 6a, when the phosphatase inhibitors were removed from lysis buffer, Y792 phosphorylation of DDR1 was completely abolished suggesting that a DDR1 tyrosine phosphatase activity was present in the lysis buffer (4–6 lanes from the left side in the left upper panel). Importantly, dapagliflozin treatment (0.5 mM, 20 min treatment) also almost completely abolished Y792 tyrosine phosphorylation of DDR1 (the furthest left lane in the left upper panel).

Next, we tested whether dapagliflozin treated (0.5 mM, 20 min treatment) lysate contains tyrosine phosphatase like activity to reduce Y792 phosphorylation of DDR1. To do this, we mixed equal amount of DMSO treated lysate and dapagliflozin (0.5 mM, 20 min) treated lysate and incubated for 30 min at room temperature (the second lane from the left side in the left upper panel). Y792 tyrosine phosphorylation of DDR1 levels were compared to either DMSO treated lysate alone (the third lane from the left side in the left upper panel) or dapagliflozin treated lysate alone sample (the most left lane in the left upper panel). As shown in Figure 6a,b, dapagliflozin treated lysate showed greater DDR1 tyrosine phosphatase activity compared to DMSO treated lysate. These results were confirmed by increased dephosphorylation of Y402 residue in Pyk2 which is downstream target of DDR1 (Figure 6c).

### 3.5. Clinical Cases of Patients with Colon, Hepatic Cell, Pancreatic, or Lung Cancer and Type 2 Diabetes Mellitus Treated with Dapagliflozin

**Figure 7:**
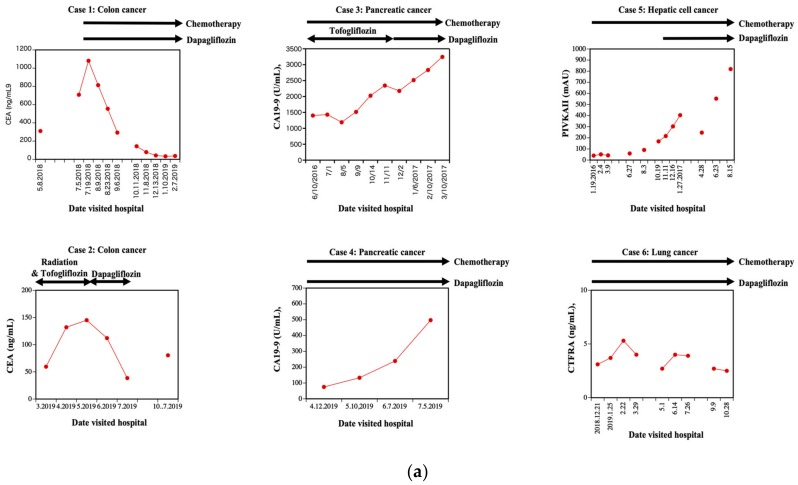

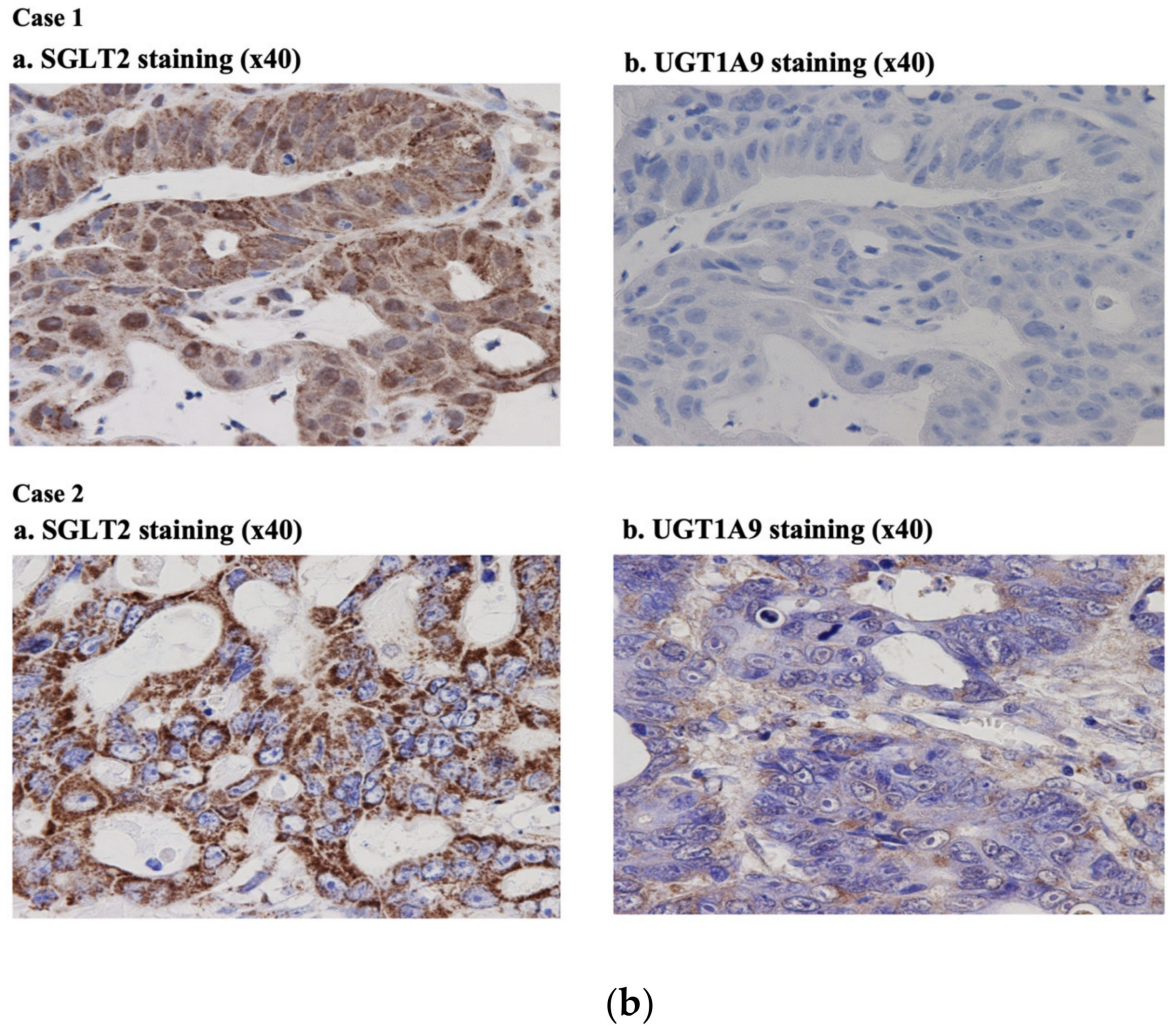
Clinical cases-Patients with type 2 diabetes mellitus and cancer were treated by sodium-glucose cotransporter 2 (SGLT2) inhibitor. (**a**) Clinical cases – Patients with type 2 diabetes mellitus and cancer treated by SGLT2 inhibitors. Six cases were demonstrated in this research. Cases 1 and 2 concern patients associated with colon cancer and type 2 diabetes mellitus. The CEA changes are presented in a line graph; chemotherapy, radiation therapy, and dapagliflozin administration are represented by black arrows (Figure 7a, case 1 and 2). In case 2, there was a period where in the patient was treated with dapagliflozin alone, and the CEA level declined. However, the CEA level started to rise after the cessation of dapagliflozin. In this patient, tofogliflozin combined with radiation therapy did not show a good effect. Cases 3 and 4 concern patients associated with pancreatic cancer and type 2 diabetes mellitus. The CA19-9 changes are presented in a line graph; chemotherapy and dapagliflozin administration are represented by black arrows (Figure 7a, case 3 and 4). Case 5 is a patient associated with liver cancer and type 2 diabetes mellitus. The PIVKAII changes are presented as a line graph; chemotherapy and dapagliflozin administration are represented by black arrows (Figure 7a, case 5). Case 6 is a patient associated with lung cancer (squamous cell carcinoma; patient experienced three separate recurrences despite dapagliflozin administration) and type 2 diabetes mellitus. The CYFRA changes are presented in a line graph; chemotherapy and dapagliflozin administration are represented by black arrows (Figure 7a, case 6). (**b**) Immunohistochemical analysis. The SGLT2 and UGT1A9 staining results of cases 1 and 2 are shown in Figure 7b. Immunostaining was performed as reported previously (16). In both cases, SGLT2 was stained well in cancer cells, but UGT1A9 was not. Please note that the material of case 1 was obtained by biopsy and the material of case 2 was obtained b.

We have identified six patients with different solid tumors and type 2 diabetes mellitus that were treated for their cancers and with the introduction of SGLT2 inhibitors to treat their diabetes. Cases 1 and 2 were patients with colon cancer and type 2 diabetes mellitus and the temporal changes in the colon cancer marker (Carcinoembryonic antigen; CEA) levels are presented in a line graph (Figure 7a, case 1, 2). Cases 3 and 4 were patients associated with pancreatic cancer and type 2 diabetes mellitus. The pancreatic cancer marker (Carbohydrate antigen 19-9; CA19-9) changes are presented in a line graph (Figure 7b, case 3, 4). Case 5 is a patient with hepatic cell cancer and type 2 diabetes mellitus. The changes in the hepatic cell cancer marker (Protein induced by vitamin K absence or antagonist II; PIVKAII) are presented as a line graph (Figure 7a, case 5). Case 6 is a patient associated with lung cancer (squamous cell carcinoma) and type 2 diabetes mellitus. The changes in the lung cancer marker (Cytokeratin 19 fragment; CYFRA) are presented in a line graph (Figure 7a, case 6). Interestingly, dapagliflozin treatment correlated with improvement in tumor burden marker for the two colon cancer patients. The SGLT2 and UGT1A9 staining results of cases 1 and 2 are shown in Figure 7b (case 1 and case 2, respectively). Immunostaining was performed as reported previously [9]. In both cases, SGLT2 staining was positive in the cancer cells, but UGT1A9 was relatively negative.

## 4. Discussion

We reported previously that dapagliflozin treatment of cultured adherent cells induced cell detachment, without affecting cell proliferation or apoptosis [9]. To more carefully examine this property of dapagliflozin, we performed time- and dose-dependent analyses on two tumor cell lines, HCT116 and HepG2 over relative short times as prolonged treatment with dapagliflozin (24–72 h) resulted in the loss of adhesion for all cells [9] and therefore analyses of the relative cell type sensitivity and molecular mechanism could not be performed.

The HCT116 cells were significantly more sensitive to dapagliflozin treatment, with cell detachment detectable by 35 min at 0.125-mM, whereas HepG2 cells were not affected up to 2-mM dapagliflozin. The higher than physiologic concentration of dapagliflozin required for the induction of HCT116 cell detachment is likely due to either the need for the actual uptake of dapagliflozin into the cells or due the relative levels of UGT1A9. Consistent with this latter possibility, UGT1A9 knockdown by shRNA in HepG2 cells rendered these cells more sensitive to dapagliflozin treatment, whereas the overexpression of UGT1A9 in HCT116 cells made them more resistant to dapagliflozin. In addition to those results, dapagliflozin did not appear to have any effect on PANC-1 and H1792 cells, likely due to the very low levels of SGLT2 expression that would prevent the cellular uptake of dapagliflozin. Similarly, as empagliflozin is degraded by four different enzymes (UGT2B7, UGT1A3, UGT1A8, UGT1A9) and tofogliflozin by five different enzymes (CYP2C18, CYP3A4, CYP3A5, CYP4A11, CYP4F3), it is not surprising that these two SGLT2 inhibitors were without affect in HCT116, HepG2, PANC-1 and H1972 cells.

Recently, the SGLT2 inhibitor canagliflozin was reported to attenuate the development of hepatocellular carcinoma in a mouse model of human NASH [17]. Additionally, SGLT2 has been reported as a diagnostic marker and therapeutic target for early-stage lung adenocarcinoma [18]. As canagliflozin is metabolized and excreted by UGT1A9 and UDP glucuronosyltransferase family 2 member B4 (UGT2B4), we speculate that cancer cells expressing SGLT2 combined with low levels of UGT1A9 and UGT2B4, will make these tumor cells sensitive to canagliflozin. Since not all SGLT inhibitors suppress the proliferation of tumor cells [19], further studies are needed to assess the anti-cancer potentials of new glucose-lowering agents. In addition, we have observed that unmetabolized dapagliflozin increased ADAM10 activity that proteolytically cleaves DDR1 resulting in the loss of cell adhesion. Whether this also underlies the anti-tumor action of canagliflozin remains to be determined.

In this study, we have demonstrated that dapagliflozin affects cellular interactions with collagens I and IV, and it is well established that DDR1 is activated by its binding to collagen I and IV and plays important role on cellular substratum adherence. In addition to the before mentioned proteolytic amino terminal DDR1 cleavage by ADAM10, dapagliflozin also induced Y792 tyrosine dephosphorylation of DDR1 leading to loss of DDR1 downstream substrate kinase activity.

Clinically, we previously reported a patient in whom SGLT2 levels were the same in a colon tumor as in the normal colon epithelium [20]. However, the levels of UGT1A9 were substantially reduced in the tumor compared to the healthy tissue. Thus, it is likely that there are individuals with discordant levels of SGLT2 and UGT1A9 levels. We have identified two more similar cases associated with type 2 diabetes and colon cancer had relatively high SGLT2 levels, but low UTG1A9 levels (Figure 7a,b, cases 1 and 2, respectively). Importantly, in case 2, there was a period wherein the patient was treated with dapagliflozin alone, and CEA level declined. However, the CEA level started to rise after cessation of dapagliflozin. In this patient, tofogliflozin combined with radiation therapy did not show improvement. Once again, these additional findings suggest that in human colon cancer, individuals with discordant levels of SGLT2 and UTG1A9 are potent candidates for dapagliflozin anticancer therapy. However, our limited patient data does suggest that dapagliflozin will not have benefit in pancreatic cancer, liver cancer, or lung cancer, consistent with our in vitro cell culture data.

The concentration of dapagliflozin which we used for our in vitro studies was higher than IC50 for patients with type 2 diabetes mellitus. Nevertheless, we observed two cases that orally administered dapagliflozin has the potential to reduce colon cancer cell volume. Interestingly, the same concentration of empagliflozin and tofogliflozin did not show any effect on colon cancer, liver, pancreatic, or lung cancer cells, suggesting that dapagliflozin’s effect was physiological. However, our current data indicated that if a high dose of dapagliflozin could be delivered directly to colon cancer tissue, rather than systemically, we would observe greater and more consistent effects of dapagliflozin as an anticancer agent. Interestingly, there are two potential approaches that could be employed, colonoscopic dapagliflozin spraying and placement of a dapagliflozin-coated stent over colon cancer lesions. By developing these approaches, we believe that we could create new translational research to contribute to the treatment of patients associated with type 2 diabetes mellitus and colon cancer.

## 5. Conclusions

We found that dapagliflozin treatment induces a loss of substratum adherence and that the sensitivity of these cells is dependent on the relative levels of the SGLT2 and dapagliflozin-inactivating protein UGT1A9. Dapagliflozin affects cellular interactions with collagens I and IV, and it is well established that DDR1 plays an important role in collagen I and IV substratum adherence. Consistent with these data, we observed that dapagliflozin treatment induced a loss of the full-length DDR1 through the activation of ADAM10 activity. Based on our clinical experience, we speculate that it is likely there are individuals with discordant levels of SGLT2 and UTG1A9 and our findings suggest that dapagliflozin could be a potential anticancer therapeutic for such patients.

## Abbreviations

DDR1	discoidin domain receptor I
ADAM10	ADAM metallopeptidase domain 10
SGLT2	sodium-glucose cotransporter 2
UGT1A9	UDP Glucuronosyltransferase Family 1 Member A9
UGT2B7	UDP Glucuronosyltransferase Family 2 Member B7
UGT1A3	UDP Glucuronosyltransferase Family 1 Member A3
UGT1A8	UDP Glucuronosyltransferase Family 1 Member A8
CYP2C18	cytochrome P450 family 2 subfamily C member 18
CYP3A4	cytochrome P450 family 3 subfamily A member 4
CYP3A5	cytochrome P450 family 3 subfamily A member 5
CYP4A11	cytochrome P450 family 4 subfamily A member 11
CYP4F3	cytochrome P450 family 4 subfamily F member 3
HEPES	4-(2-hydroxyethyl)-1-piperazineethanesulfonic acid
CEA	Carcinoembryonic antigen
CA19-9	Carbohydrate antigen
PIVKAII	Protein induced by vitamin K absence or antagonist II
CYFRA	Cytokeratin 19 fragment
